# The interaction between health and personal anxiety in children

**DOI:** 10.1192/j.eurpsy.2021.1229

**Published:** 2021-08-13

**Authors:** I. Shishkova, E. Pervichko

**Affiliations:** 1 Faculty Of Clinical Psychology, Ryazan State Medical University named after I.P. Pavlov, Ryazan, Russian Federation; 2 Faculty Of Psychology, Lomonosov Moscow State University, Moscow, Russian Federation; 3 Faculty Of Psychology And Social Sciences, Pirogov Russian National Research Medical University, Moscow, Russian Federation

**Keywords:** Short Health Anxiety Inventory, Health anxiety in children, health psychology, Mental health prevention in children

## Abstract

**Introduction:**

In modern health psychology there is a question of separating the concepts of “personal anxiety” and “health anxiety” and defining the interaction features and mutual influence between these concepts.

**Objectives:**

To study the interaction between personal anxiety and health anxiety in children, taking into account the parents’ influence and depending on the child’s personal illness experience.

**Methods:**

The sample: 145 respondents (46 frequently ill children (mean age 16.3±0.3), 41 rarely ill children (mean age 16.1±0.1), 28 parents of frequently ill children (mean age 44.9±0.8), 30 parents of rarely ill children (mean age 44.5±1.5)). We used: “Short Health Anxiety Inventory” (SHAI; Salkovskis et al., 2002), STAI (Spielberger, 2002).

**Results:**

We find significant differences in the personal anxiety indicator (1.386, p≤0.01), which is higher in frequently ill children (moderate level of severity). Parents of frequently ill children have the same level of personal anxiety (no statistically significant differences) (12.825, p>0.05). For groups of rarely ill children and their parents we find significant differences (2.382, p≤0.01), and the level of personal anxiety is higher in children. The indicator of health anxiety in frequently and rarely ill children has no significant differences (9.265, p>0.05). The same is typical for rarely ill children and their parents while in the groups of frequently ill children and their parents this indicator has significant differences and is higher in parents (9.136, p ≤0.01).

**Conclusions:**

The results show that health anxiety is an independent construct, the consideration of which should begin with non-clinical, normative forms of manifestation in both adults and children.

